# Increment of Access Points in Integrated System of Wavelength Division Multiplexed Passive Optical Network Radio over Fiber

**DOI:** 10.1038/srep11897

**Published:** 2015-07-08

**Authors:** I. S. Amiri, S. E. Alavi, M. R. K. Soltanian, N. Fisal, A. S. M. Supa’at, H. Ahmad

**Affiliations:** 1 Photonics Research Centre, University of Malaya, 50603 Kuala Lumpur, Malaysia; 2 UTM MIMOS CoE in Telecommunication Technology, Faculty of Electrical Engineering, Universiti Teknologi Malaysia, 81310 UTM Johor Bahru Malaysia; 3 Lightwave Communication Research Group, Faculty of Electrical Engineering, Universiti Teknologi Malaysia, 81310 UTM Skudai, Johor, Malaysia

## Abstract

This paper describes a novel technique to increase the numbers of access points (APs) in a wavelength division multiplexed-passive optical network (WDM-PON) integrated in a 100 GHz radio-over-fiber (RoF). Eight multi-carriers separated by 25 GHz intervals were generated in the range of 193.025 to 193.200 THz using a microring resonator (MRR) system incorporating an add-drop filter system. All optically generated multi-carriers were utilized in an integrated system of WDM-PON-RoF for transmission of four 43.6 Gb/sec orthogonal frequency division multiplexing (OFDM) signals. Results showed that an acceptable BER variation for different path lengths up to 25 km was achievable for all four access points and thus the transmission of four OFDM channels is feasible for a 25 km standard single mode fiber (SSMF) path length.

Increasing demands for video-based interactive and multimedia services drive requirements for very large bandwidths and high data rates in next-generation access networks. In this regard, the 100 GHz radio over fiber (RoF) system with an associated low cost and high transmission performance is introduced as a promising technique to satisfy the bandwidth requirements for delivering multi Gb/s services to substantial numbers of users in optical and wireless access systems^1^. Using RoF technology allows for the possibility to replace expensive broadband electronics with all-optical signal processing functions, one example being optical millimeter wavelength (MMW) generation in base stations (BSs). However, a major disadvantage of 100 GHz MMW signals lies in access convergence being limited to short distances of less than 10 meters due to high atmospheric loss^2^,^3^. Apart from RoF, cost-effective wavelength division multiplexed-passive optical networks (WDM-PON) are able to support several local subscribers in wired access networks. WDM-PON has several advantages such as large data bandwidth (high capacity), large coverage range, enhanced security, upgradeability, and scalability^4^,^5^,^6^.

In order to increase the capacity and coverage area of the 100 GHz RoF access networks, integration with WDM-PON provides a very appealing solution such that the integrated system of WDM-PON-RoF can be considered as an auspicious scheme to distribute wired and wireless services simultaneously. Several recent reports on WDM-PON-RoF systems were mainly focused on different means of modulation^7^,^8^,^9^,^10^,^11^,^12^, or they are only designed for the downstream link, i.e. without upstream link, in an ROF system^13^,^14^.

While such innovations have enhanced the integrated system, less effort has been spent on providing more access points (APs) for users in optical network units (ONUs) since the number of ONUs is limited in a PON^15^. In the WDM-PON-RoF architecture, the number of APs that can be provided is based on optical multi-carrier generation in optical line terminal (OLT). There have been several techniques reported up to now for the generation of multi-carriers used in optical communications^6^,^9^,^10^,^11^,^16^. One method to generate optical multi-carriers lies in adjusting parameters of Mach-Zehnder modulators (MZM) such as biasing point^17^,^18^,^19^.

Microring resonators (MRRs) have attracted considerable attention in the field of optical communications^20^,^21^. In addition to the superior stability and beam quality associated with muti-carriers generated by MRRs, a chief objective of using MRR systems lies in producing greater numbers of multi-carriers than have been previously reported. Achieving such an objective will lead to an increase in serviceable APs and hence a dramatic upsurge in the capability of entire integrated systems^22^,^23^,^24^,^25^,^26^.

Optical MRR, filters and switches have been successfully demonstrated in the two important material systems comprising GaAs-AlGaAs and GaInAsP-InP^27^,^28^,^29^,^30^, and^31^ reports the fabrication process of the vertically-coupled InP devices. Generation of optical multi-carriers is possible via MRR systems, wherein nonlinear light behavior occurs inside an MRR after it receives a strong pulse of light input^32^,^33^,^34^. Ring resonators can be used as filter devices whereby suitable selection of system parameters leads to generation of high frequency (GHz) carrier signals^35^. The add-drop ring resonator system in connection with other ring resonators can be used to generate the optical soliton pulses of GHz frequency that are necessary for wired and wireless optical communication^26^,^36^. The advantage of the proposed system is that the transmitter can be fabricated on-chip or operated alternatively by a single device. Exciting new technological progress, particularly in the field of tunable narrow band laser systems, multiple transmission, and MRR systems, provides the foundation for the development of new transmission techniques. In addition to improvements in efficiency and beam quality, these soliton sources provide better quality transmission^37^,^38^,^39^.

Although it is possible to generate great numbers of optical carriers using MRRs, in this study, eight optical carriers are generated at the OLT using the MRR system. In order to achieve high spectral efficiency (SE) and better transmission performance multi-carrier modulation schemes such as frequency division multiplexing signal (OFDM) is used. Hence, four of these carriers are used to modulate 43.6 Gb/s OFDM for downlink to provide wired and wireless connections, and the other four carriers remain un-modulated for use in generating a 110 GHz OFDM signal. The unmodulated carriers are also split to be employed for uplink connection via a wavelength reuse technique. Theoretical analysis indicates this scheme has excellent performance and constitutes a promising candidate for future hybrid access networks.

## Theoretical background

**Figure 1:**
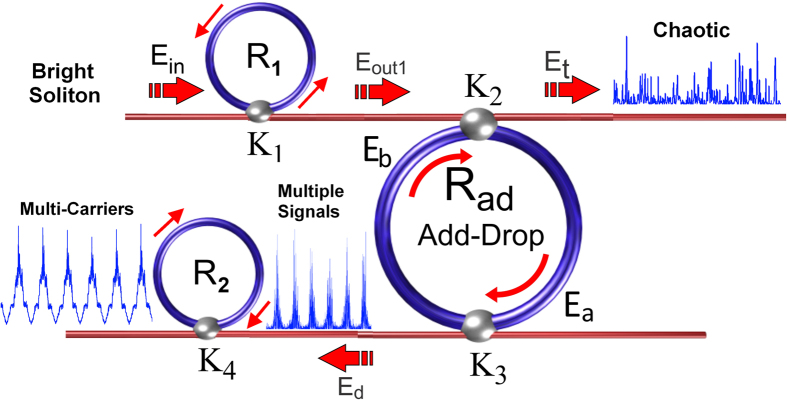
MRR system, R: ring radii, κ: coupling coefficients, R_ad_: add-drop ring radius, E_in_: input power, E_out_: Ring resonator output E_t_: throughput output, E_d_: drop port output, E_a_ and E_b_: Circulating fields.

The proposed system of THz frequency band generation is shown in Fig. 1. Here, a series of MRRs are incorporated to an add-drop filter system. The filtering process of the input soliton pulse is performed via the MRRs, in which frequency bands ranging from 193.025 to 193.200 THz can be obtained. Soliton self-phase modulation provides for a large output gain that is necessary in order to balance the dispersion effects of the linear medium. It is important to note that soliton solutions of the nonlinear wave equation are temporally very stable. Solitons are also highly stable against changes of the properties of a medium, provided that these changes occur over sufficiently long distances, and as such, solitons can adiabatically adapt their shape in response to the slowly-varying parameters of a medium^40^.

Ring resonators are made from waveguides by a fabrication process in which the construction medium has the Kerr effect-type nonlinearity. This Kerr effect causes the refractive index (n) of the medium to vary in accordance with^41^


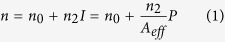


where *n*_0_ and *n*_2_ are the linear and nonlinear refractive indices respectively, and *I* and *P* are the optical intensity and power respectively. The effective mode core area, *A*_*eff*_, ranges from 0.10 to 0.50 μm^2^ in terms of material parameters for InGaAsP/InP^42^. A bright soliton with a 1.55 μm central frequency and 800 mW power is introduced into the first ring resonator, *R*_1_. The input optical field *E*_*in*_ of the optical bright soliton is given by^43^


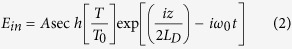


Where *A* and *z* are the amplitude of the optical field and propagation distance respectively, *L*_*D*_ is the dispersion length of the soliton pulse, and *ω*_*0*_ is the carrier frequency of the signal. The soliton pulse maintains temporal width invariance while it propagates and is hence known as a temporal soliton. A balance of *L*_*D*_ = *L*_*NL*_ should be achieved between the dispersion length *L*_*D*_ and the nonlinear length 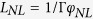, where Γ = *n*_2_ × *k* is the length scale over which disperse or nonlinear effects make the beam become wider or narrower. The normalized output of the light field, which is the ratio between the output and the input fields for each round trip, can be expressed by^44^


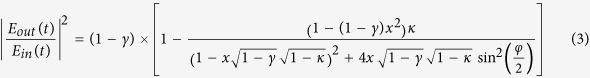


*κ* is the coupling coefficient and *x* = exp(−*αL*/2) represents the round trip loss coefficient, where the ring resonator length and linear absorption coefficient are given by L and α respectively. *ϕ* = *ϕ*_0_ + *ϕ*_*NL*_, where *ϕ*_0_ = *kLn*_0_ and 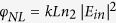 are the linear and nonlinear phase shifts respectively. The wave propagation number in vacuum and the fractional coupler intensity loss are given by *k* = 2*π*/*λ* and *γ* respectively^45^. The bright soliton pulse is input into the nonlinear MRRs, whereupon a chaotic signal can be formed via selection of appropriate parameters. For the add-drop system, the interior electric fields *E*_*a*_ and *E*_*b*_ are expressed as^46^


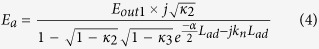



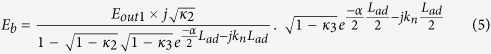


Where *κ*_2_ and *κ*_3_ are the coupling coefficients, *L*_*ad*_ = 2*πR*_*ad*_, and *R*_*ad*_ is the radius of the add-drop system. The throughput and drop port electrical fields of the add-drop system can be expressed as^47^


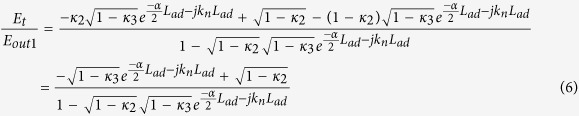



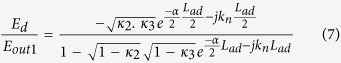


The normalized optical outputs of the ring resonator add-drop system can be expressed by equations (8) and (9).


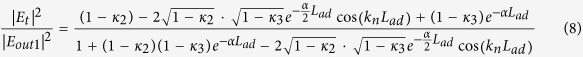



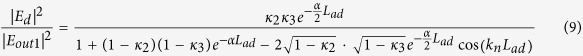


where 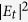 and 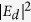 are the optical output powers of the through and drop ports respectively^48^.

## Results of soliton generation

**Figure 2:**
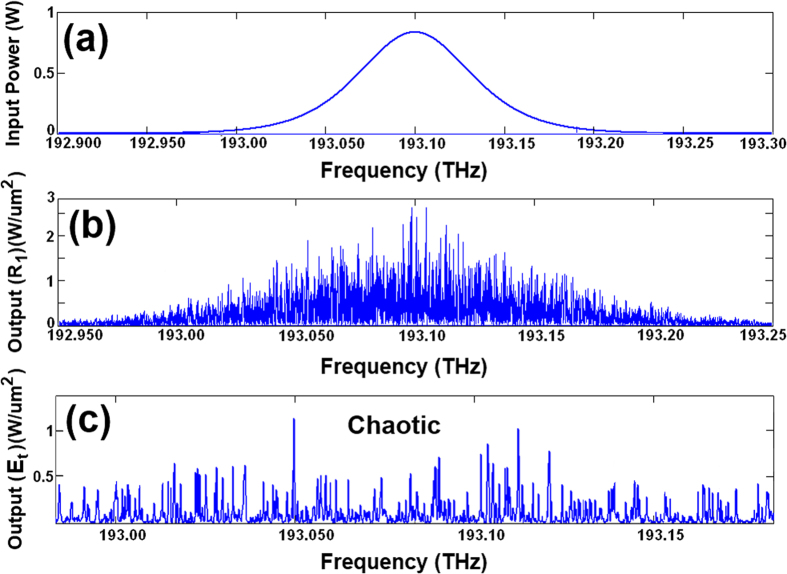
Results of soliton signals: (**a**) input bright soliton, (**b**) output from first MRR which is input into the add-drop MRR, and (**c**) throughput output signals from the add-drop MRR.

The parameters of the MRR system are listed in Table 1. The results of the chaotic signal generation are shown in Fig. 2. The input pulse of the bright soliton pulse with a power of 800 mW, as represented by Fig. 2(a), was inserted into the system. A bright soliton pulse inserted into the nonlinear system was chopped into smaller signals spreading over the spectrum, and the subsequent nonlinear effects resulted in a large bandwidth arising in the MRRs. Careful selection of ring parameters resulted in the creation and trapping of a frequency soliton pulse within the system.

**Figure 3:**
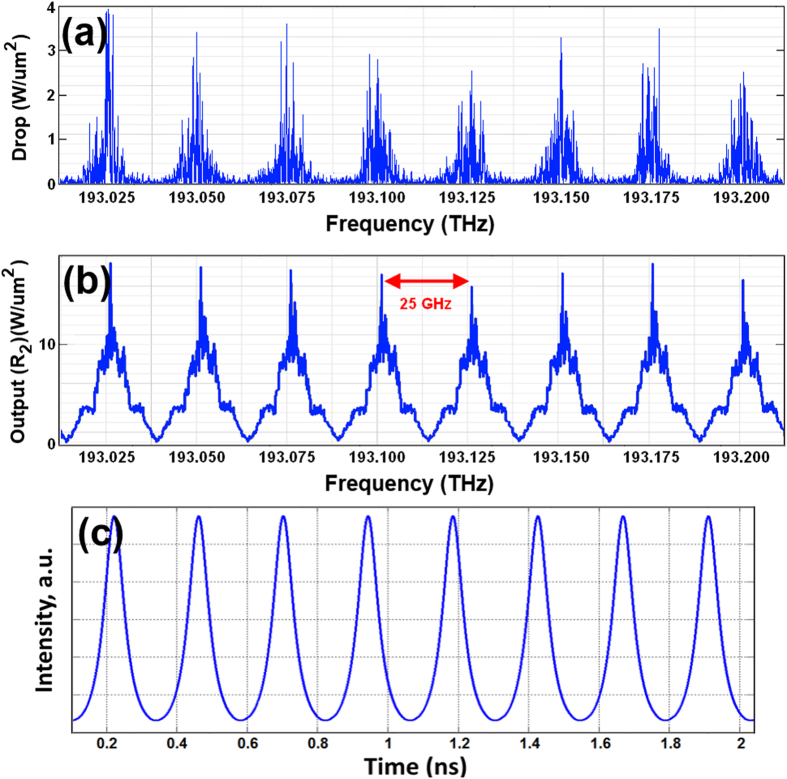
Results of multi-carrier signal generation, (**a**) output signal from the drop port of the add-drop MRR (*R*_*ad*_), (**b**) multi-carrier output signals from the MRR (*R*_*2*_), and (**c**) time-domain waveform of the signal.

Chaotic soliton pulses are widely used as carrier signals in securing optical communication, wherein information is inserted within the signals and later retrieved via appropriate filtering systems wherein a filtering of the carrier signal occurs as pulses pass through the MRRs. The output signal from the MRR (*R*_*1*_), as shown in Fig. 2(b), was inserted into the add-drop ring resonator. The throughput output signals from the add-drop MRR can be seen in Fig. 2(c), where soliton pulses range from 193 to 193.15 THz. The drop port of the system showing multiple chaotic signals as illustrated in Fig. 3(a). These signals could be filtered within the MRR (*R*_*2*_) during the round-trips, thus allowing clear carrier signals ranging from 193.025 to 193.200 THz to be generated as represented in Fig. 3(b). The free spectral range (FSR) of these signals was calculated as FSR = 25 GHz. Figure 3(c) shows the signal in time domain.

One important aspect of the system is that carrier signals possessing desired parameters, e.g. for full width at half maximum (FWHM), can be obtained at the drop port of the add-drop filter system by tuning parameters of the system such as the ring radius and coupling coefficients. The soliton pulses are sufficiently stable that their shape and velocity are preserved during travel along the medium. In addition to improvements in efficiency and beam quality, these soliton sources also provide better quality transmission as reported in^49^.

## System setup

**Figure 4:**
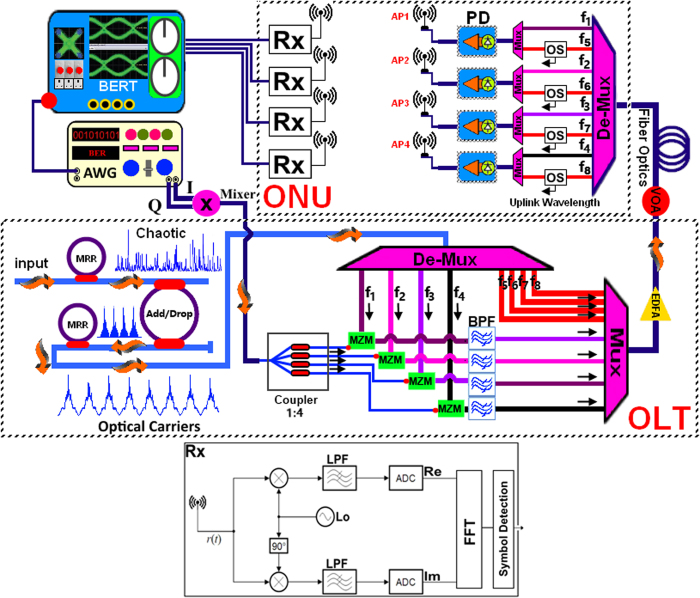
System setup.

The schematic of the system setup is shown in Fig. 4. At the OLT, the MRRs were connected to an add-drop MRR in order to generate multi-carriers. As described in the previous section, eight optical carriers of different wavelengths were generated using the MRR system. These carriers are labeled as f_1_….f_8_. The carriers are first separated via de-multiplexer and four of them (f_1_…f_4_) are modulated using the MZM while the rest (f_5_…f_8_) are kept un-modulated.

Data transmission with OFDM technique has been done by Kaur *et al*^50^. As shown in Fig. 2 (a) in the manuscript, the input bright soliton has a Gaussian wide spectrum. By changing the MRR’s radius, the longer or shorter FSR could be achieved in the drop port^51^, i.e. the MRR could filter out some specific wavelengths. These extracted wavelengths corresponded to f_1_,…, f_8_ in the frequency domain. Four carriers, of frequency f_1_ to f_4_, were used to transmit four continuous OFDM signals optically via MZM; continuous waves of OFDM signals were generated from an arbitrary waveform generator (AWG) as possesses the parameters listed in Table 2. A power coupler with 1:4 ratio was utilized to enable generation of four separate continuous OFDM signals.

The length of the OFDM symbol signal is equal to


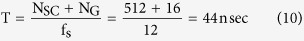


The data rate for the 16-QAM-OFDM signals is equal to:


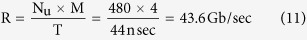


Here, M is the number of bits per symbol. This generated baseband OFDM signal is moved to IF region using an I/Q mixer with f_IF_ = 10 GHz. The 1:4 RF power coupler is used to generate four OFDM signals.

**Figure 5:**
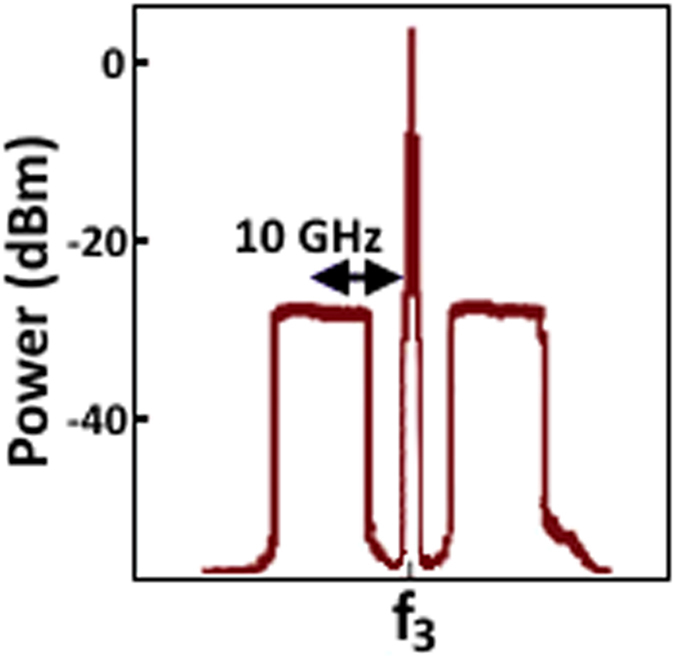
Modulated subcarrier with OFDM signal.

The modulated carrier e.g f_3_ has two sidebands, as can be seen in Fig. 5, in which the four band-pass filters (BPFs) were used to suppress the carrier and the upper sideband of each modulated signal to generate the optical single sideband (OSSB) without carrier signals. Each BPF has the center wavelength (CWL) and the bandwidth (BW) as specified below:


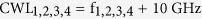



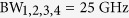


Subsequently, the four OSSB signals and the four un-modulated carriers were multiplexed, amplified by an erbium doped fiber amplifier (EDFA), and the consequent signal was transmitted through 25 km standard single mode fiber (SSMF).

**Figure 6:**
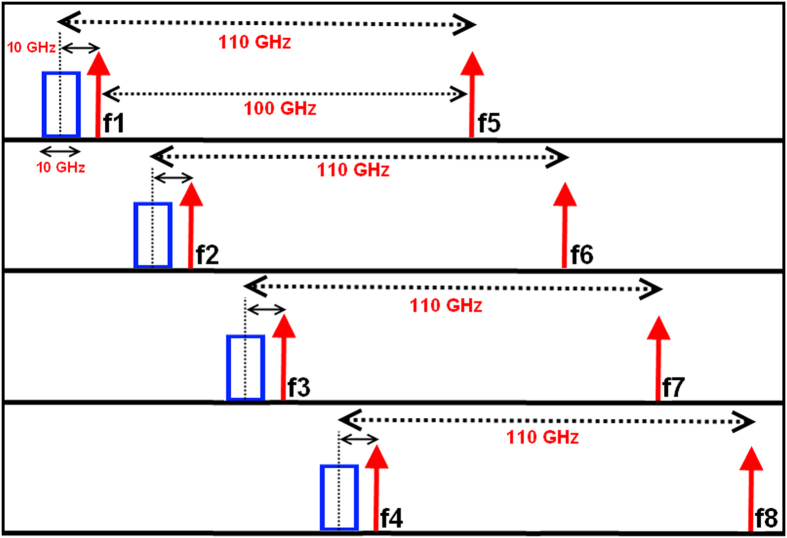
W-band signals generation.

At the ONU section, the de-multiplexer was used to receive a beam consisting of multiple optical frequencies from an SMF and subsequently separate it into frequency components. Here, as shown in Fig. 6, the first channel was multiplexed with the un-modulated wavelength f_5_ which had a 100 GHz frequency difference, the second channel was multiplexed with f_6_, and so on. For APs, the un-modulated carriers were split into two parts by an optical splitter (OS) and frequency centered at the frequency of an un-modulated carrier to be used for uplink applications.

Accordingly, there were four APs (AP1…AP4), in which high-speed photo diodes (PDs) are used to generate four electrical 110 GHz mm-wave signals for wireless propagation via the transmitter antenna. To transmit and receive W-band signals, a dielectric resonator array antenna was used. The array antenna is mainly used to increase the antenna gain. It is noted that the antenna gain is stable and exceeds 23 dBi in all operational bands. At the receiver side the propagated W-Band signal is received by the OFDM receiver (Rx) and is demodulated and detected using local oscillator (LO) with W-band frequency. The detected signal is further analyzed with bit error rate tester (BERT).

**Figure 7:**
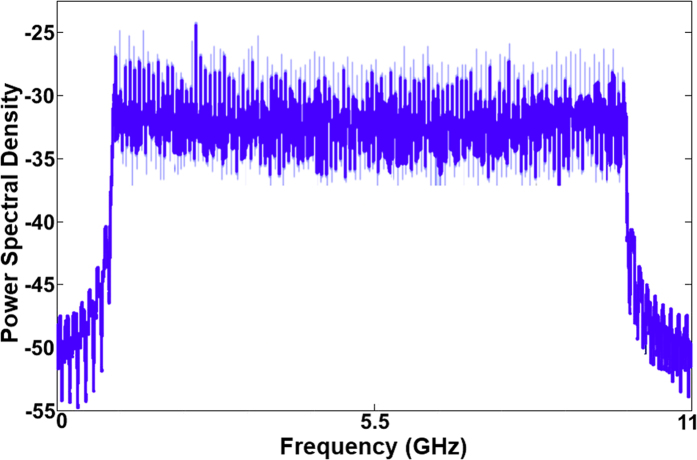
Received OFDM signal for AP1.

In order to investigate the optical link performance, the total optical power level after amplification was adjusted with a variable optical attenuator (VOA) from −3 to 7 dBm. A propagated RF signal was received at the receiver antenna base station of each AP, with a corresponding received signal to AP1 having the spectrum pattern representative of that shown in Fig. 7.

**Figure 8:**
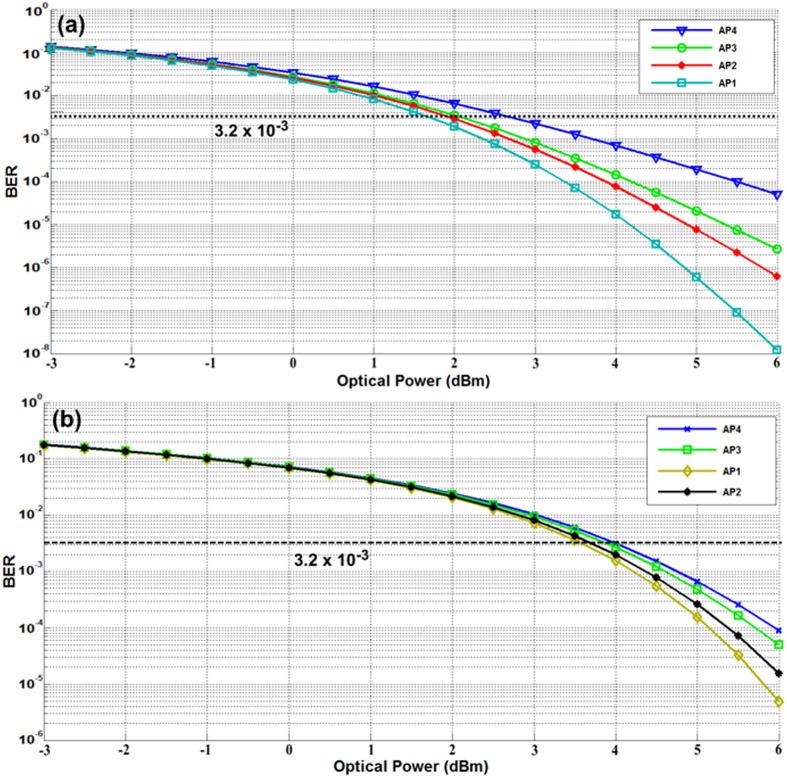
System performance under two optical lengths, (**a**) B2B, and (**b**) 25 Km.

At the receiver side the detected signal was analyzed in order to evaluate the bit error rate (BER) of each AP, where BER measurement provides for an assessment of digital communication signal quality and evaluation of link degradation. The BER results for APs at different optical power and in different optical path lengths (back-to-back (B2B) and 25Km) are shown in Fig. 8. The 3.2 × 10^−3^ BER is the threshold for successful transmission and is represented in the figure by a dashed line.

**Figure 9:**
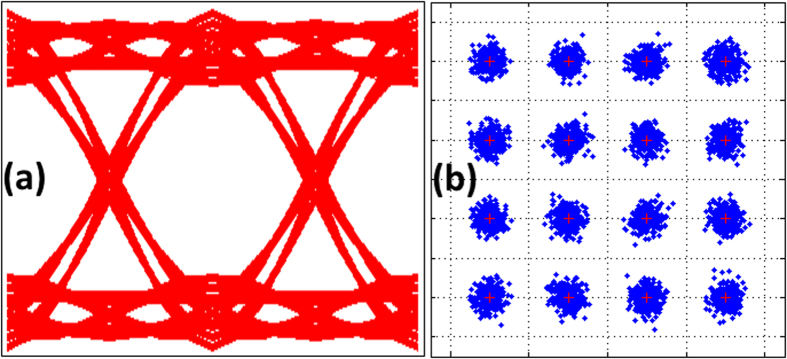
AP1 performance, (**a**) eye diagram, and (**b**) constellation diagram.

As illustrated in Fig. 8, the system performance for two circumstances, 25 km fiber link and B2B, was investigated. In consideration of the BER threshold of 3.2 × 10^−3^, it can be concluded from observation of the figure that increasing the received power of all APs allows for acceptable BER performance. For the B2B case, the minimum optical power to achieve successful transmission to all APs was 2.7 dBm, while minimum optical power was 4 dBm for 25 km fiber; this latter increase mostly resulted from the dispersion effects of the SMF link. Figure 9 shows the system performance in the case of AP1 servicing when the wireless distance is 2 m and the optical link is 25 km. Observation of the eye and constellation diagrams presented in this figure led to a conclusion that it is possible to use MRR to generate multi-carriers with suitability for WDM-PON-ROF applications.

**Figure 10:**
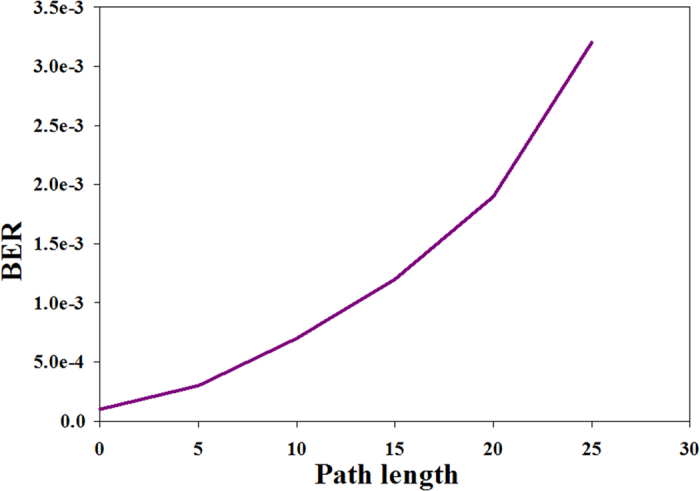
BER vs. different path lengths at an optical power of 4 dBm.

A graph of BER vs. different path lengths at an optimal optical power value of 4 dBm for one particular access point (AP3) is illustrated in Fig. 10.

The BER value increased, as shown in Fig. 10, as a consequence of dispersion effects in the fiber optic component. The achieved optimum path length was 25 km for an optical power of 4 dBm.

## Conclusion

A MRR system incorporating an add-drop filter system was used to generate a THz frequency band. Filtering of the input pulse within the system resulted in the generation of multi-carriers suitable for use in WDM-PON-RoF communication. Eight multi-carriers separated by 25 GHz intervals were generated in a frequency range spanning 193.025 to 193.200 THz by using MRRs. All these optically generated multi-carriers were used in RoF applications. Results provided evidence that the proposed system allows for four access points to be serviced with an acceptable BER variation for different path lengths. This paper shows the transmission of four OFDM channels is feasible for up to a 25 km SMF path length and should be of considerable interest as a basis for further work in this area.

## Additional Information

**How to cite this article**: Amiri, I. S. *et al.* Increment of Access Points in Integrated System of Wavelength Division Multiplexed Passive Optical Network Radio over Fiber. *Sci. Rep.* **5**, 11897; doi: 10.1038/srep11897 (2015).

## Figures and Tables

**Table 1:** Fixed parameters of the MRR system.

*R*_ad_	*R*_1_	*R*_2_	*κ*_1_	*κ*_2_	*κ*_3_	*κ*_4_
100 μm	10 μm	5 μm	0.5	0.7	0.5	0.9
*n*_0_	*n*_2_ (m^2^W^−1^)	*A*_eff1_ (μm^2^)	*A*_eff2_ (μm^2^)	*α* (dBmm^−1^)	*γ*	
3.34	2.2 × 10^–17^	0.50	0.25	0.5	0.1	

**Table 2:** OFDM parameters.

OFDM parameters	Value
Sampling rate	f_s_ = 12 G Samples/Sec
IFFT size	N_SC_ = 512
Number of modulated subcarriers	N_u_ = 480
Cyclic prefix	N_G_ = 16
IF Frequency	10 GHz
